# Spinal Epidural Abscess in Melioidosis: A Rare Case Report from Eastern India

**DOI:** 10.7759/cureus.4187

**Published:** 2019-03-06

**Authors:** Vinay Prabhat, Amrit Gantaguru, Sudarsan Behera, Rajesh Rana, Gurudip Das

**Affiliations:** 1 Orthopaedics, All India Institute of Medical Sciences, Bhubaneswar, IND; 2 Orthopedics, All India Institute of Medical Sciences, Bhubaneswar, IND

**Keywords:** spinal epidural abscess, melioidosis, paraparesis

## Abstract

A spinal epidural abscess (SEA) is a rare condition that has a devastating impact on the patient’s health. It is difficult to diagnose and can present with a myriad of symptoms with or without the involvement of a neurological deficit. The conditions that lead to immunocompromised status, such as malnutrition, diabetes, intravenous drug abuse, previous surgical intervention, and human immunodeficiency virus (HIV) infection/acquired immune deficiency syndrome (AIDS) can predispose a patient to SEA. The most common organisms isolated from the affected patient include Staphylococcus aureus and Streptococcus species while an abscess in some cases can be caused by tuberculosis and fungal and parasitic infections. Among the other causative organisms is Burkholderia pseudomallei (B. pseudomallei), also known as Pseudomonas pseudomallei, which is a Gram-negative, bipolar, aerobic, motile, and rod-shaped bacterium. It is a soil-dwelling bacterium, which is endemic in tropical and subtropical regions worldwide, particularly in Thailand and northern Australia, and causes melioidosis. To our knowledge, SEA caused by B. pseudomallei from the Indian subcontinent has not been reported in the literature. In this case report, we present the case of a patient with SEA caused by B. pseudomallei.

## Introduction

Spinal epidural abscess (SEA) is a rare but potentially devastating condition, which may lead to a permanent neurological deficit and even death if not managed promptly. It is caused by an infection of the soft tissues and bone of the spinal column characterized by the accumulation of pus in the epidural space, which leads to spinal cord compression. The incidence of SEA has been increasing in the last two-three decades, from 0.2-1 case to 2.5-3 cases per 10,000 hospital admissions [1]. Usually, SEA patients present with a complaint of mild back pain and fever before the onset of acute neurological deterioration [2-4]. However, the classical triad of fever, back pain, and neurologic deficit is found in only 10%-15% of patients with SEA who present to the physician for the first time [2-3]. If the diagnosis is delayed, the further evolution of symptoms occurs in four phases: (1) localized spinal pain; (2) radicular pain and paresthesia; (3) muscular weakness, sensory loss, and sphincter dysfunction; and (4) paralysis [4]. Only a few cases of SEA with paraplegia have been reported till date [5]. SEA with paraplegia is an emergency condition that requires the urgent decompression of the spinal cord, as it may lead to a permanent neurological deficit if left untreated. The pathophysiology involved in the neurological deterioration in SEA mainly includes mechanical obstruction and pressure necrosis [6].

The most common causative organism of SEA is Staphylococcus aureus while other organisms, such as Escherichia coli, coagulase-negative staphylococci, Bacteroides species and anaerobes, Pseudomonas species and Streptococci, including Streptococcus viridans, group B streptococci, and pneumococcus also cause this condition [1,7-8]. To our knowledge, SEA caused by Burkholderia pseudomallei from the eastern region of the Indian subcontinent has not been reported. B. pseudomallei is a Gram-negative bacillus, which is endemic in the tropical and subtropical regions but is usually not endemic to India. It is the causative agent of melioidosis and causes abscesses in the lung, liver, spleen, parotid glands, and skeletal muscles [9-11]. Patients with immunocompromised status, such as those with diabetes mellitus, chronic renal failure, and alcoholic cirrhosis, are more susceptible to SEA [12-13]. In this case report, we present a rare case of SEA with paraparesis caused by Burkholderia in a patient with type II diabetes. The patient initially presented with low back pain mimicking sacroiliitis, but later, the condition was diagnosed as SEA and managed with midline posterior decompression and evacuation of pus along with antibiotics.

## Case presentation

**Figure 1:**
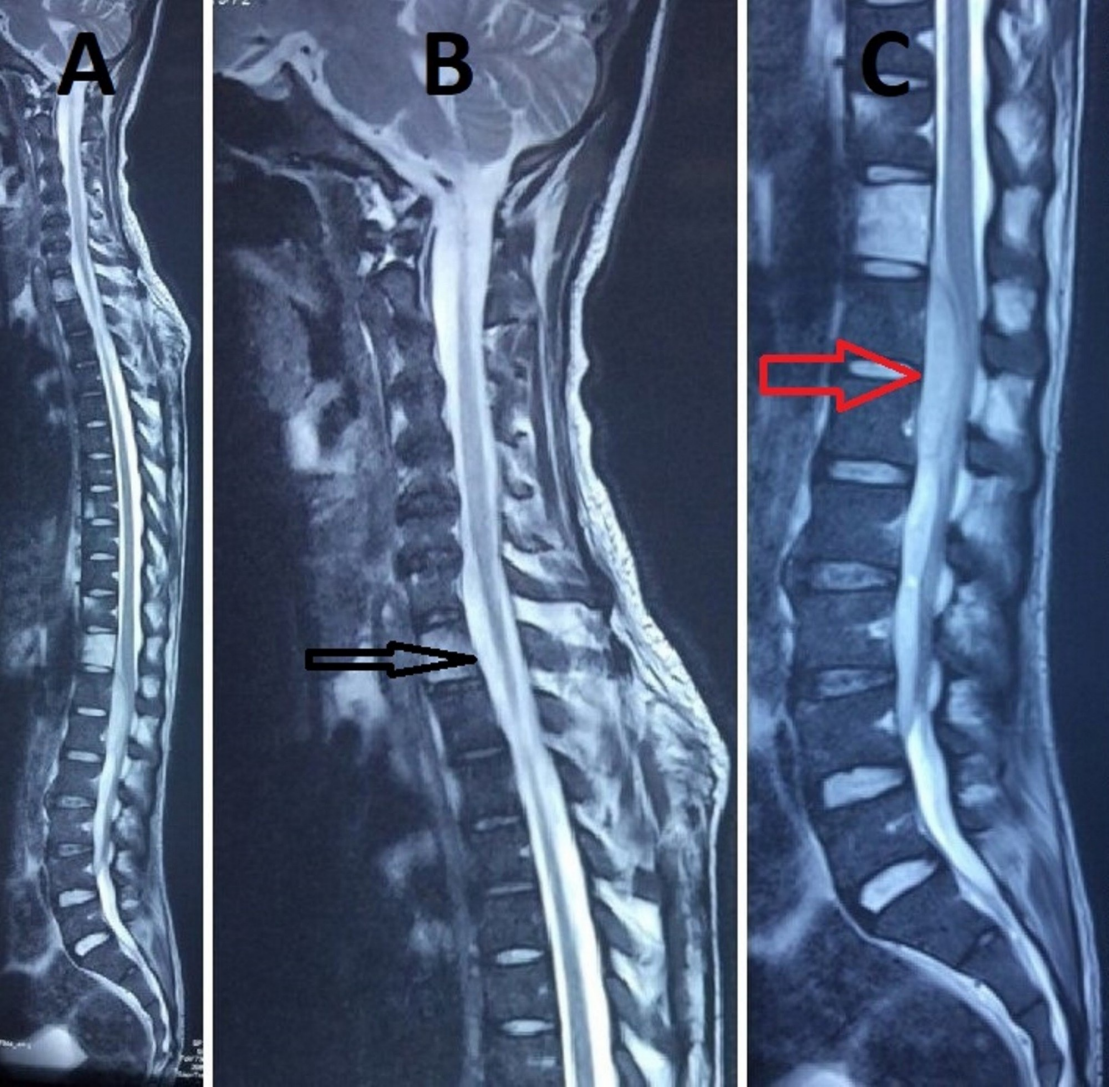
Magnetic resonance imaging (MRI) of whole spine (sagittal section) 1A: MRI of whole showing lesion in the cervical and lumbar region 1B: MRI of the cervical spine showing epidural abscess (C_5_-T_5_) (arrow mark) 1C: MRI of the dorsolumbar spine showing epidural abscess (D_12_-L_4_) (arrow mark) MRI: magnetic resonance imaging

**Figure 2:**
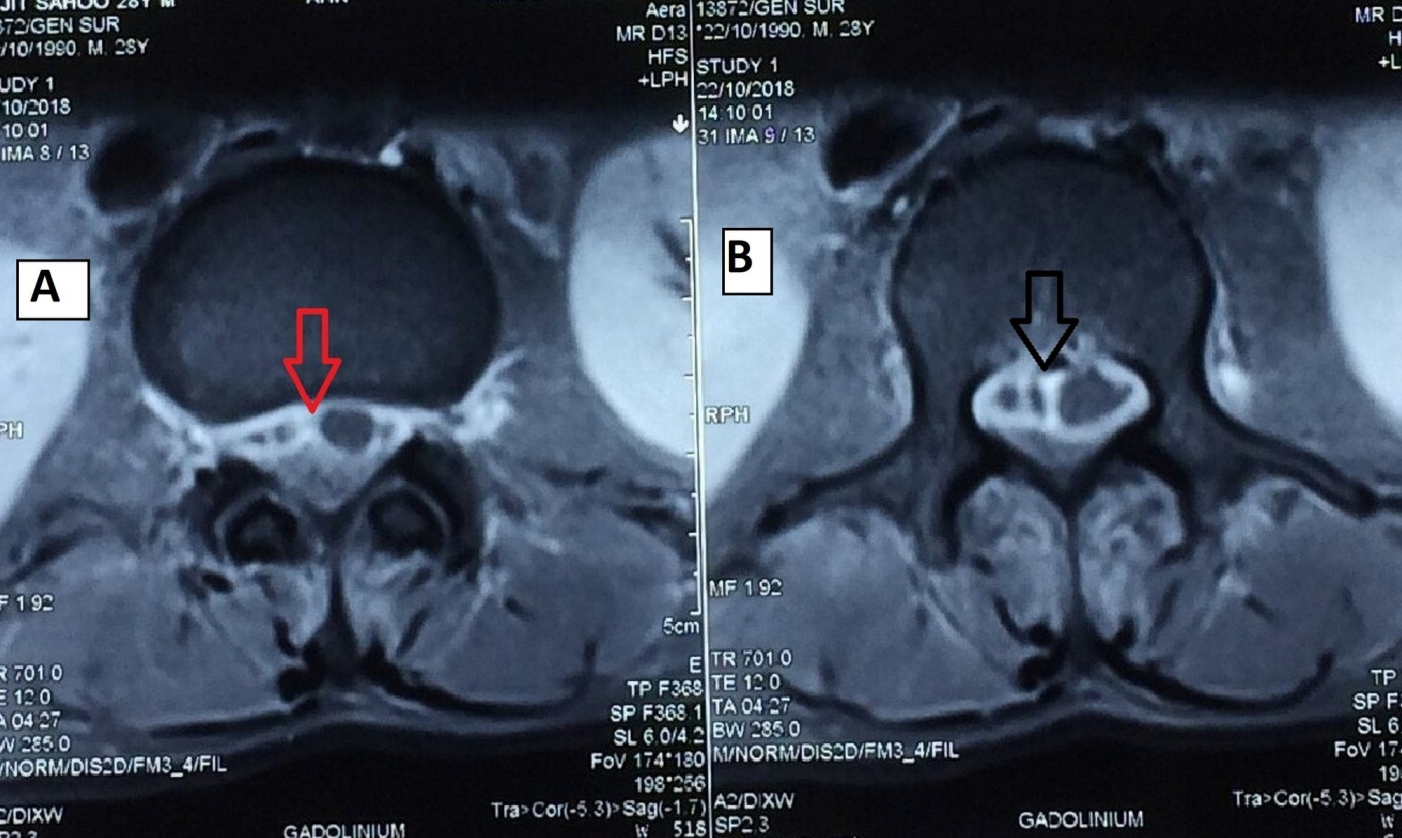
A and B: MRI (axial section) showing a lobulated abscess in the epidural space (arrow mark) MRI: magnetic resonance imaging

A 28-year-old male patient presented in casualty with complaints of fever with chills and rigors with pain in the abdomen for a period of 20 days. The patient had initially taken antipyretics and antibiotics but found no effect. On clinical examination, the patient was febrile, and his abdomen was soft without any guarding or rigidity. Routine investigation showed that the patient had diabetes, which was uncontrolled. The test results indicated that the patient also had a high erythrocyte sedimentation rate along with a high total leukocyte count and a high level of positive C-reactive protein. Therefore, insulin and empirical antibiotics were started immediately. An ultrasonographic examination of the abdomen revealed hepatomegaly, chronic pancreatitis, and splenomegaly with multiple splenic abscesses. An endoscopic examination of the upper gastrointestinal tract showed the presence of esophageal candidiasis with a dilated vein at the fundus. Contrast-enhanced computed tomography (CECT) of the abdomen indicated chronic calcific pancreatitis with splenic vein thrombosis, multiple splenic abscesses with evidence of rupture and ascites, and bilateral pleural effusion with basal lung collapse. Blood culture report showed the presence of B. pseudomallei, which was sensitive to imipenem and aminoglycosides, and so the patient was put on antibiotics. A few days through the course of treatment, the patient complained of low back pain. Clinical examination revealed tenderness at the bilateral sacroiliac joints with no neurological deficit. Plain radiographic examination confirmed the diagnosis of bilateral sacroiliitis and, therefore, twice-daily sulfasalazine 500 mg was added to the treatment regimen. One week later, the patient presented again with unimproved back pain and paraesthesia and weakness in both the lower limbs. On musculoskeletal examination, the power of both the lower limbs was found to be 4/5, with decreased sensation in the bilateral L_4_, L_5_, and S_1_ dermatomes. An examination of the upper limb showed no neurological deficit. Magnetic resonance imaging (MRI) of the spine showed abnormal hyper-intensity of C_5_, D_1_, D_8_, and D_12_ vertebra body with post-contrast non-enhancement of the portion of D_12_ vertebra along with anterior epidural, left paraspinal, left iliac, and sacroiliac abscesses (Figures 1A-1C and Figures 2A-2B).

**Figure 3:**
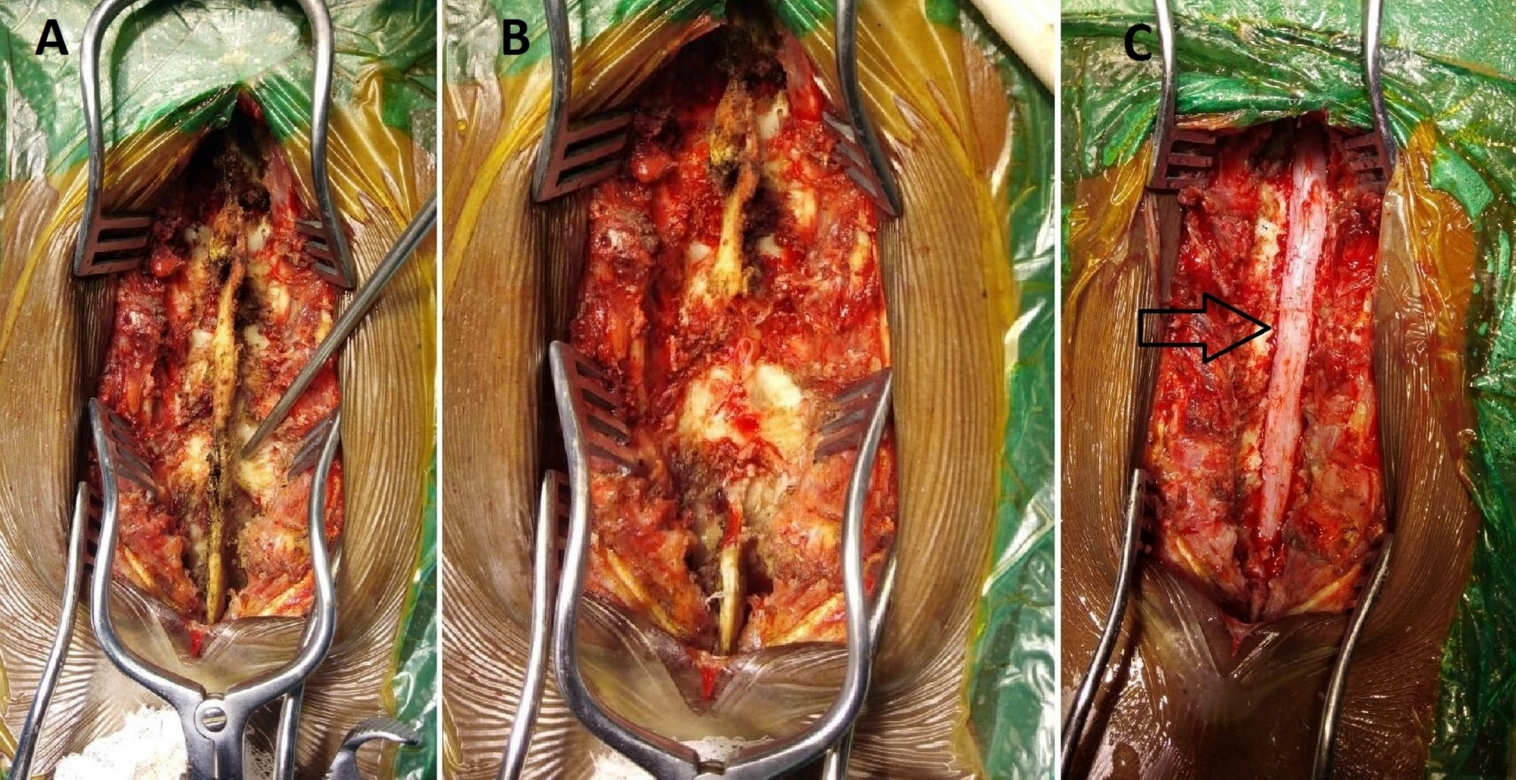
Intraoperative clinical photographs 3A: A clinical photograph showing D11–L5 3B: Removal of the spinous process from D12 to L4 3C: A clinical image of the spinal cord (arrow) after decompression

Bilateral sacroiliitis was also confirmed by MRI examination, so emergency decompression was planned for the patient. Accordingly, a laminectomy was performed, extending from D_12_ to L_4_ vertebra without affecting facet joints and the intraoperative abscess was found to be loculated (Figures 3A-3C).

Proper decompression with an evacuation of pus was done, and the obtained pus was sent to the laboratory for culture and sensitivity analysis. The culture and sensitivity report was suggestive of the growth of B. pseudomallei, which was sensitive to imipenem and aminoglycosides. Postoperatively, the neurological status of the patient improved, and he was shifted to the general surgery unit where he was operated for a splenic abscess. Intravenous antibiotics were continued for six weeks, and the patient was discharged. At the three-month follow-up, the patient had recovered completely.

## Discussion

SEA usually presents as fever and back pain, and if the diagnosis is delayed, the symptoms evolve into paraplegia [2-4]. The patient who presented to us had fever, back pain, and abdominal pain initially, and after 25 days of initial presentation, he developed symptoms of neurological deficit. The diagnosis of SEA was delayed, as its incidence is very rare and the classical triad of fever, back pain, and neurological deficit occurs in only 10%-15% of all those who present with SEA for the first time [1-3]. MRI is usually performed to best visualize the location and extent of abscesses, including those involving spinal, paraspinal, and disc regions, as compared to other imaging techniques, such as CT myelography [14]. In our case, the MRI examination revealed a hyperintensity of the C_5_, D_1_, D_8_, and D_12_ vertebra body with an anterior epidural abscess from D_12_ to L_4_ and C_5_ to D_1_, along with left paraspinal, left iliac, and sacroiliac paraspinal abscesses. The clinical symptoms of the patient suggested paraplegia due to abscess from D_12_ to L_4_, but no neurological signs suggestive of abscess in the cervical region were seen. As early intervention gives better results, surgical decompression was planned for the abscess in the dorsolumbar region at the earliest [15-16]. As soon as the patient was admitted, empirical antibiotics were started based on the blood culture and sensitivity report. Decompression and evacuation of pus were done following laminectomy from D_12_ to L_4_, as the abscess was found to be loculated and thus could not be drained at a single level, but the facet joint was preserved to maintain stability (Figure 3) [17]. Unlike a single-level spinal dural abscess, which could be managed by percutaneous CT-guided aspiration with antibiotics, a multiple-level abscess, which is lobulated, needs open decompression [18]. The patient was treated with parenteral antibiotics for six weeks followed by treatment with oral antibiotics for six weeks based on the sensitivity report.

The most common causative organism of SEA is S. aureus [1,7-8], but in our case, the causative organism was found to be B. pseudomallei, which was detected preoperatively in blood culture and in pus, which was obtained during drainage and cultured for sensitivity analysis. B. pseudomallei is a Gram-negative bacillus that causes abscesses in the liver, lungs, spleen, parotid glands, and skeletal muscles in immunocompromised patients, including those with diabetes, chronic kidney disease, and alcoholic cirrhosis living in the endemic areas in East Asia, especially Thailand and northern Australia [9-13]. Although India is not an endemic area, our patient had uncontrolled diabetes when he presented to us with an abscess in the spleen, liver, and spinal epidural space. Probably, this is the first case report of SEA with paraparesis caused by B. pseudomallei.

## Conclusions

SEA is an uncommon condition and should be considered a differential diagnosis in patients presenting with fever and back pain so that it can be diagnosed early and treated promptly to prevent a permanent neurological deficit. Our case showed that though India is not an endemic country for B. pseudomallei, this organism may cause SEA in immunocompromised patients.
